# Extracellular Adhesive Cues Physically Define Nucleolar Structure and Function

**DOI:** 10.1002/advs.202105545

**Published:** 2022-02-05

**Authors:** Oscar J. Pundel, Liisa M. Blowes, John T. Connelly

**Affiliations:** ^1^ Centre for Cell Biology and Cutaneous Research 4 Newark Street London E1 2AT UK

**Keywords:** biomechanics, keratinocyte, mechanotransduction, nucleolus, nucleus

## Abstract

Adhesive cues from the extracellular matrix (ECM) specify the size and shape of the nucleus via mechanical forces transmitted through the cytoskeleton. However, the effects of these biophysical stimuli on internal nuclear architecture and cellular responses remain poorly understood. This study investigates the direct impact of ECM adhesion on nucleolar remodeling in human keratinocytes using micropatterned substrates. Limited adhesion on small micropatterns promotes fusion of nucleoli, alongside a reduction in nuclear volume and condensation of heterochromatin. These changes in nucleolar architecture are mediated by altered chromatin biomechanics and depend on integration of the nucleus with the actin cytoskeleton. Functionally, nucleolar remodeling regulates ribogenesis and protein synthesis in keratinocytes and is associated with specific transcriptional changes in ribogenesis genes. Together, these findings demonstrate that cell shape and nuclear morphology control nucleolar structure and function and implicate the nucleolus as a key mechano‐sensing element within the cell.

## Graphical abstract

This study presents new insight into the biomechanical regulation of nucleolar remodeling. The authors demonstrate that extracellular adhesive cues define the structure and function of the nucleolus via physical regulation of nuclear architecture and chromatin condensation.

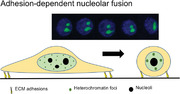

## 1 Introduction

Biophysical cues, such as changes in extracellular matrix (ECM) structure and rigidity, are sensed through the cell's intricate network of adhesion receptors and cytoskeletal elements, which in turn regulate a wide range of cellular responses and signaling cascades.^[^ ^1^ ^]^ In addition, the cytoskeleton controls nuclear architecture and chromatin remodeling through direct physical anchorage to the linker of nucleoskeleton and cytoskeleton (LINC) complex.^[^ ^2^, ^3^ ^]^ Consisting of nesprins and Sun‐domain proteins, the LINC complex spans the nuclear membrane and interacts with the nuclear lamina. The nuclear lamins act as internal scaffolding, providing mechanical integrity to the nucleus and regulation of chromatin organization.^[^ ^4^, ^5^ ^]^ Force‐induced deformation of the nucleus and chromatin remodeling mediates a wide range of cellular functions, such as DNA damage responses^[^ ^6^, ^7^, ^8^ ^]^ and cell fate decisions.^[^ ^9^, ^10^, ^11^ ^]^ Additionally, mechanical alteration of the nuclear membrane can influence cytoplasmic‐nuclear transport.^[^ ^12^ ^]^

Within the nucleus, the nucleolus is the largest organelle and a central hub for transcription of ribosomal DNA (rDNA) and regulation of ribogenesis. It is a dense, membrane‐less structure composed of rRNA, transcriptional machinery, and structural proteins.^[^ ^13^ ^]^ Nucleoli assemble through liquid–liquid phase separation processes that are thermodynamically driven and depend on the biophysical properties of both the nucleolus and surrounding chromatin.^[^ ^14^, ^15^ ^]^ As a central regulator of ribogenesis, the nucleolus controls the critical downstream functions of translation and protein synthesis, which impact fundamental cellular processes, such as growth, differentiation, and survival.^[^ ^13^ ^]^ In addition, interactions between the nucleolus and rDNA at specific nucleolar organizer regions (NORs) can influence nearby genome organization and gene expression.^[^ ^16^ ^]^ Thus, changes in nucleolar structure could have significant effects on numerous cellular functions including both metabolic and transcriptional responses. While the size and shape of nucleoli are known to change during cellular senescence or in response to stressors, such as DNA damage,^[^ ^13^ ^]^ the biophysical basis of these relationships is poorly understood. Moreover, the contribution of extracellular biomechanical cues to nucleolar regulation is unknown.

To gain new insight into the impact of biomechanical cues on the nucleolus, we employed micropatterned surfaces, which are well established tools to precisely control the available adhesive area, cytoskeletal tension, focal adhesion assembly, and cell morphology,^[^ ^17^ ^]^ and with this system, we investigated the interplay between chromatin remodeling and nucleolar organization in primary human keratinocytes (HKs). Under conditions of limited ECM adhesion, we observed striking remodeling of the nucleoli, with associated changes in heterochromatin, gene expression, and ribogenesis. Nucleolar remodeling also scaled directly with nuclear size across a wide range of adhesive cues, including ECM rigidity and cell–cell adhesion, and this response was mechanically regulated by linkage to the actin cytoskeleton and by chromatin mobility. Thus, these findings implicate the nucleolus as a previously unidentified mechano‐sensing element within the cell.

## 2 Results

### 2.1 Limited ECM Adhesion Promotes Fusion of Nucleoli

**Figure 1:**
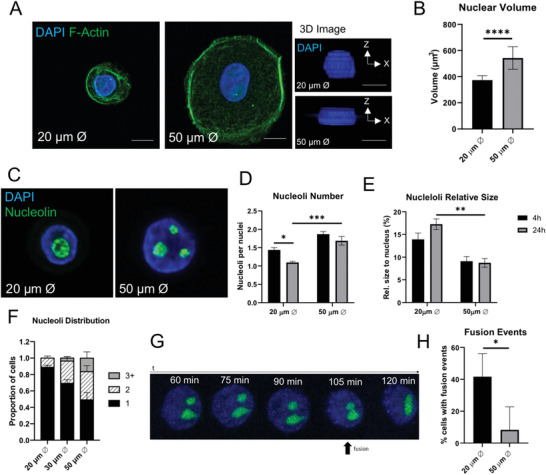
Cell‐ECM adhesion regulates nuclear size and nucleolar remodeling: A) Representative confocal immunofluorescence images (2D and 3D rendering) of HKs grown on micropatterned surfaces for 24 h, stained for nucleus (DAPI, blue) and F‐Actin (green). Scale bars = 10 µm. B) Quantification of nuclear volume shown as mean ± SEM, *N* = 3 experiments. ^****^ *p*‐value < 0.0001 (two‐tail *t*‐test). C) Representative confocal immunofluorescence images of HKs grown on micropatterned surfaces for 24 h, stained for nucleus (DAPI, blue) and nucleolin (green). Scale bars = 10 µm. D) Nucleoli number and E) relative size to the nucleus shown as mean ± SEM, *N* = 3 experiments. ^*^ *p*‐value < 0.05, ^**^ *p*‐value < 0.01, and ^***^ *p*‐value < 0.001 (2‐Way ANOVA, Tukey multiple comparisons test). F) Quantification of proportion of cells with 1, 2, or 3+ nucleoli after 24 h shown as mean ± SEM, *N* = 3 experiments. G) Representative images of nucleoli fusing in HaCaT keratinocytes transfected with Nucleolin‐GFP seeded on 20 µm islands. H) Percentage of cells with observed fusion events shown as mean ± SEM, *N* = 3 experiments. ^*^ *p*‐value < 0.05 (two‐tail *t*‐test).

To investigate the influences of cell‐ECM interactions on nucleolar remodeling, we first characterized the size and shape of nucleoli in primary HKs exposed to defined adhesive cues from our micropatterned ECM model^[^ ^17^, ^18^ ^]^ by confocal microscopy. HKs were cultured on micropatterned collagen islands of 20 or 50 µm diameter, which forced cells to adopt a round or spread morphology (**Figure** **1**A; Figure S1A, Supporting Information). Consistent with our previous findings,^[^ ^18^ ^]^ limited adhesion on the 20 µm substrates resulted in reduced nuclear volume (Figure 1B) and nuclear cross‐sectional area (Figure S1B, Supporting Information) compared to spread cells on the 50 µm islands. Reduced nuclear volume corresponded with striking changes in organization of the nucleoli on small micropatterns. HKs on 20 µm islands displayed a significant reduction in the average number of nucleoli per cell after 24 h, while HKs on 50 µm islands retained 1–3 nucleoli per nucleus (Figure 1C,D). In conjunction with the reduced nuclear volume, nucleoli on the small micropatterns occupied a higher comparative size of the nucleus compared to cells on large islands (Figure 1E). Further analysis of the distribution of nucleoli numbers, including an intermediate micropattern diameter of 30 µm, revealed that reduced nucleoli on the smaller islands reflected a progressive shift to more cells with one nucleolus and fewer cells with two, three, or more nucleoli (Figure 1F). Through time‐lapse confocal imaging of cells expressing GFP‐nucleolin, we confirmed that the reduced number of nucleoli on the small micropatterns resulted from nucleoli fusing together (Figure 1G,H). Interestingly, we observed fewer nucleoli on small islands to be a generalized phenomenon across different cell lines, indicating the potential universality of this effect (Figure S1C, Supporting Information). These findings therefore indicate that cell adhesive area regulates structural remodeling of nucleoli in HKs.

### 2.2 Keratinocyte Adhesion Regulates Lamin A/C and Chromatin Remodeling

**Figure 2:**
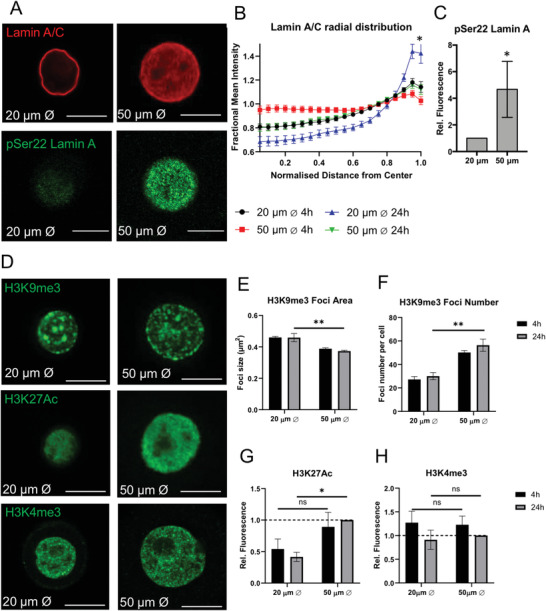
Cell‐ECM adhesion regulates Lamin A/C and chromatin remodeling: A) Representative confocal immunofluorescence images of HKs grown on micropatterned surfaces for 24 h, stained for Lamin A/C (red) and pSer22 Lamin A (green). Scale bars = 10 µm. B) Radial distribution of Lamin A/C plotted as mean fractional intensity (mean fluorescence intensity = 1) as a function of radial position within the nucleus. Data represent mean intensity profiles ± SEM of 3 experiments. ^*^ *p* < 0.05 (Kolmogorov‐Smirnov test compared to 50 µm 24 h) C) Quantification of pSer22 Lamin A levels shown as mean ± SEM of 3 experiments. ^*^ *p* < 0.05 (two tail *t*‐test). D) Representative confocal fluorescence images of histone modifications H3K9me3, H3K27Ac, and H3K4me3 (green). E,F) Analysis of H3K9me3 foci size and number shown as mean ± SEM, *N* = 3 experiments. ^**^ *p*‐value < 0.01. (2‐Way ANOVA, Tukey multiple comparisons test). G,H) Normalized integrated intensity measurements of histone modifications shown as mean ± SEM, *N* = 3 experiments. n.s: non‐significant. ^*^ *p*‐value < 0.05. (2‐Way ANOVA, Tukey multiple comparisons test).

To gain further insight into the relationship between nucleolar remodeling and nuclear architecture, we next investigated the effects of HK adhesion on the nuclear lamina and chromatin remodeling. After 24 h on micropatterned substrates, lamin A/C redistributed toward the nuclear periphery in HKs grown on small islands compared to large ones (**Figure** **2**A,B), while the overall levels did not change (Figure S2A,B, Supporting Information). Increased nucleoplasmic lamin A/C on the 50 µm micropatterns corresponded with a significantly higher level of phosphorylated (Ser 22) lamin A (Figure 2A,C), suggesting that the redistribution of A type lamins reflects changes in lamin solubility.^[^ ^19^ ^]^ In comparison, there were no differences in the radial distribution of lamin B1 and no differences in expression of LAP2*α*, which is known to interact with nucleoplasmic lamin A/C (Figure S2B,C, Supporting Information).^[^ ^19^ ^]^

Global changes in chromatin remodeling were analyzed by expression of key histone modifications. We observed the heterochromatin marker H3K9me3 associating into larger and less numerous foci in HKs grown on small islands (Figure 2D–F; Figure S2D, Supporting Information). Levels of the open chromatin marker H3K27Ac were also significantly reduced in HKs grown on small islands after 24 h, while there were no differences in H3K4me3, a marker of active transcription (Figure 2D,G,H). Further analysis of the spatial distribution of these chromatin marks within the nucleus revealed that H3K27Ac and H3K9me3 were concentrated near the periphery of the nucleus but did not differ between micropatterns (Figure S2E,F, Supporting Information). Together, these results demonstrate that reduced nuclear volume in HKs is associated with redistribution of lamin A/C to the nuclear periphery, condensation of heterochromatin foci, and reduced levels of open chromatin.

### 2.3 Keratinocyte Adhesion Regulates Defined Transcriptional Programs

**Figure 3:**
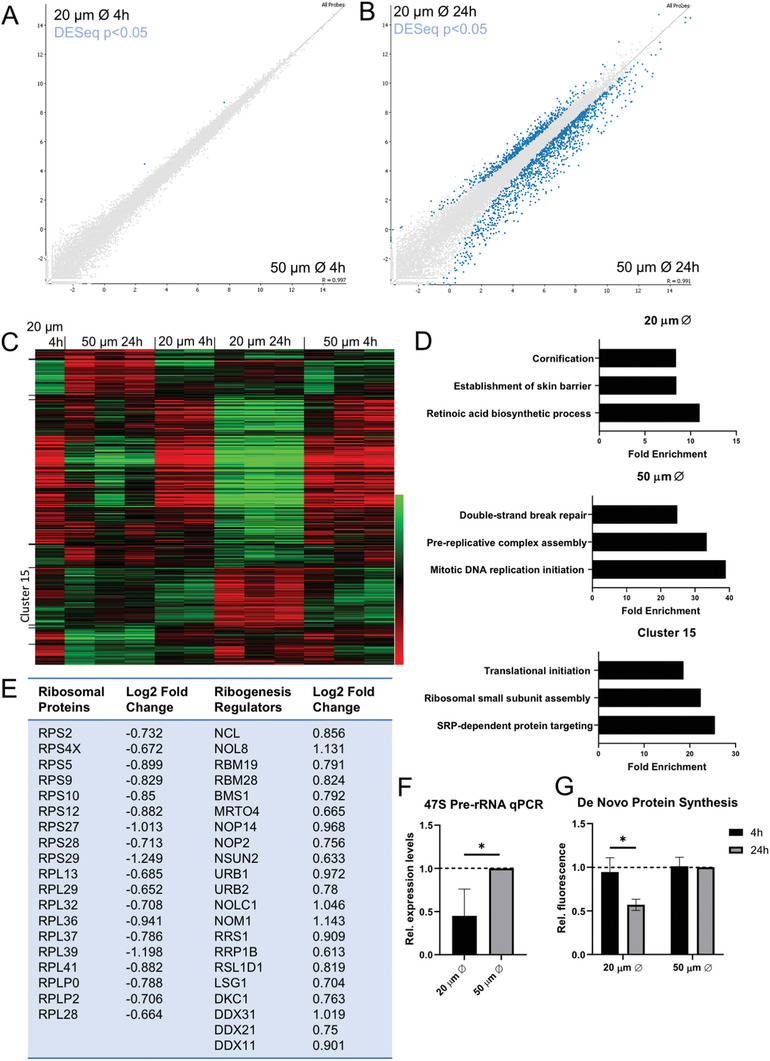
Cell‐ECM adhesion regulates distinct patterns of gene expression: A,B) Scatterplot of differentially expressed genes between HKs cultured on 20 and 50 µm islands at 4 and 24 h. Highlighted in blue are DESeq2 significantly differentially expressed genes (alpha 0.05). C) Unsupervised hierarchical clustering plot of all samples with highlighted cluster of interest (cluster 15). D) Three most enriched GO terms of genes upregulated on 20 µm, upregulated on 50 µm, and within cluster 15. E) Differential expression of genes associated with the ribogenesis pathway in HKs grown on micropatterned islands after 24 h expressed as Log2 (transcripts on 50 µm islands/transcripts on 20 µm islands). F,G) Relative expression levels of 47S pre‐ribosomal RNA as quantified from qPCR and de novo protein synthesis levels as quantified by normalized integrated intensity measurements of epifluorescence microscopy images of nascent proteins. Data represent mean ± SEM, *N* = 3 experiments. ^*^ *p*‐value < 0.05 (two‐tail *t*‐test for 47S qPCR and 2‐way ANOVA, Tukey multiple comparisons test for de novo protein synthesis).

To determine the downstream transcriptional responses associated with altered adhesion and nuclear remodeling, we next analyzed changes in the transcriptional profile of HKs cultured on micropatterned substrates. Next‐generation RNA sequencing (RNA‐seq) was performed on HKs cultured on 20 or 50 µm islands for 4 or 24 h. Analysis of differentially expressed genes revealed only two significantly different genes at the 4 h time point (**Figure** **3**A), indicating initially similar patterns of gene expression on the two island sizes. After 24 h, over 1500 genes were differentially expressed between HKs on 20 and 50 µm islands (Figure 3B; Dataset S1, Supporting Information). Principle components analysis and hierarchical clustering identified several clusters of genes to be significantly different between island sizes at 24 h (Figure 3C; Figure S3A, Supporting Information). As expected, genes associated with terminal differentiation were enriched on the small micropatterns, while proliferation associated genes were enriched on the large micropatterns (Figure 3D). Gene ontology analysis further revealed genes associated with retinoic acid (RA) biosynthesis to be upregulated on small patterns, and DNA damage response genes upregulated on the large patterns (Figure 3D). In addition, there was a cluster of up and down regulated genes (cluster 15), associated with ribogenesis, translation, and protein secretion (Figure 3D). Several differentially expressed genes from each pathway were selected and verified by qPCR (Figure 3B).

DNA damage response genes were upregulated in HKs grown on large islands. Double strand break repair sites were analyzed by imaging of co‐localized *γ*H2AX and 53BP1 foci and confirmed that cells on large islands were undergoing more DNA repair than cells on small islands (Figure S3C,D, Supporting Information). By contrast, the RA biosynthesis pathway was upregulated on small islands, and consistent with these changes, transcriptional activity of an RA response element reporter was elevated on small islands (Figure S3E, Supporting Information).

Given the effects of HK adhesion on nucleolar remodeling, the changes in the ribogenesis pathway were particularly intriguing. Here, we observed a downregulation of ribogenesis regulatory genes in HKs cultured on small islands, while genes for ribosomal proteins were upregulated (Figure 3E), possibly indicating a compensation mechanism. To assess the functional impact on ribogenesis, we next analyzed the expression of 47S pre‐ribosomal RNA levels by qPCR as it is the initial rRNA transcribed from rDNA and further processed into ribosomes. De novo protein synthesis was also measured by pulsed labeling with a methionine analog. Limited adhesion on the small micropatterns significantly reduced both 47S rRNA expression and protein synthesis (Figure 3F,G). These results demonstrate that the transcriptional changes in gene expression induced by altered HK‐ECM adhesion are associated with key cellular functions, including DNA damage repair, RA signaling, and ribogenesis. Moreover, nucleolar fusion on the small islands corresponded with altered expression of ribogenesis genes, reduced transcription of rRNA, and reduced protein synthesis, suggesting a potential functional link between nucleolar architecture and ribogenesis.

### 2.4 Nuclear Volume Defines Nucleolar Structure and Function

**Figure 4:**
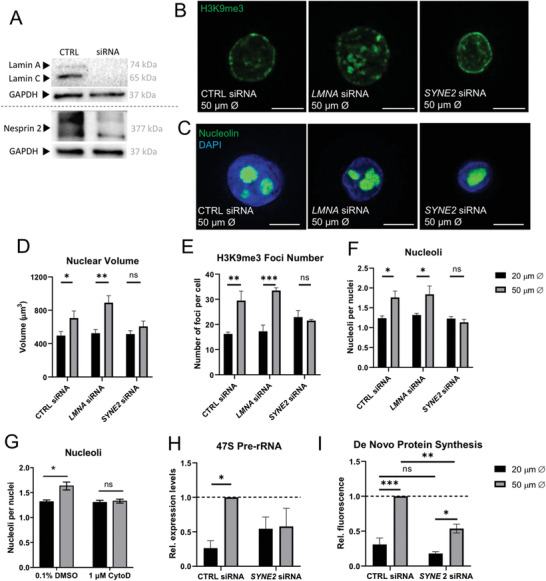
Nesprin‐2 mediates adhesion‐dependent changes in nuclear volume and nucleoli organization: A) Western blot of lamin A/C and Nesprin‐2 after siRNA treatment of HKs compared to non‐targeting control (CTRL) siRNA. Blots are representative of *N* = 2 experiments. B) Representative confocal immunofluorescence images of primary HKs grown on micropatterned surfaces for 24 h and treated with CTRL, *LMNA*, or *SYNE2* siRNA, stained for H3K9me3 (green). Scale bars = 10 µm. C) Representative confocal immunofluorescence images of primary HKs grown on micropatterned surfaces for 24 h and treated with CTRL, *LMNA*, or *SYNE2* siRNA, stained for nucleus (DAPI, blue) and nucleolin (green). Scale bars = 10 µm. D) Quantification of nuclear volume, E) number of H3K9me3 foci, and F) number of nucleoli from immunofluorescence images. Data represent mean ± SEM, *N* = 3 experiments. n.s: non‐significant. ^*^ *p*‐value < 0.05, ^**^ *p*‐value < 0.01, and ^***^ *p*‐value < 0.001 (2‐Way ANOVA, Tukey multiple comparisons test). G) Quantification of nucleoli number in HKs cultured on micropatterns for 24 h and treated with carrier (0.1% DMSO) or 1 *μ* m cytochalasin D. Data represent mean ± SEM, *N* = 3 experiments. ^*^ *p*‐value < 0.05 (2‐Way ANOVA, Tukey multiple comparisons test) H) Quantification of 47S pre‐ribosomal RNA and I) de novo protein synthesis levels in HKs treated with CTRL or *SYNE2* siRNA and cultured on micropatterns for 24 h. Data represent mean ± SEM, *N* = 3 experiments. n.s: non‐significant. ^*^ *p*‐value < 0.05, ^**^ *p*‐value < 0.01, and ^***^ *p*‐value < 0.001 (2‐Way ANOVA, Tukey multiple comparisons test).

To further investigate the biophysical relationship between nuclear architecture and ribogenesis we next examined the specific roles of the nuclear lamina and the mechanical linkage to the actin cytoskeleton. We used siRNA to knock down *LMNA* and *SYNE2* genes, which code for lamin A/C and nesprin‐2, respectively (**Figure** **4**A). While lamin A/C is essential for the mechanical integrity of the nucleus,^[^ ^20^ ^]^ nesprin‐2 is the primary mechanical linkage between the nuclear membrane and the actin cytoskeleton in keratinocytes.^[^ ^21^ ^]^ While *LMNA* siRNA treatment did not significantly alter nuclear size, *SYNE2* knockdown significantly reduced nuclear volume of HKs on large islands, similar to the nuclear volume of cells cultured on small islands (Figure 4B,C), suggesting that forces from the actin cytoskeleton mediated nuclear expansion on the large islands. In addition, the number of heterochromatin foci and the number of nucleoli scaled with nuclear size, as seen by reduced H3K9me3 foci and number of nucleoli in HK only with *SYNE2* knockdown on 50 µm micropatterns (Figure 4B–F). The role of the actin cytoskeleton was also confirmed by treatment with the F‐actin depolymerizing agent cytochalasin D, which blocked the effects of micropattern size on the number of nucleoli (Figure 4G). *SYNE2* siRNA treatment also blocked micropattern‐dependent 47S rRNA transcription and reduced overall protein synthesis levels (Figure 4H,I). We therefore conclude that adhesion‐induced changes in nuclear volume are mediated by nesprin‐2 linkage to the actin cytoskeleton and define nucleolar structure, ribogenesis, and protein synthesis.

**Figure 5:**
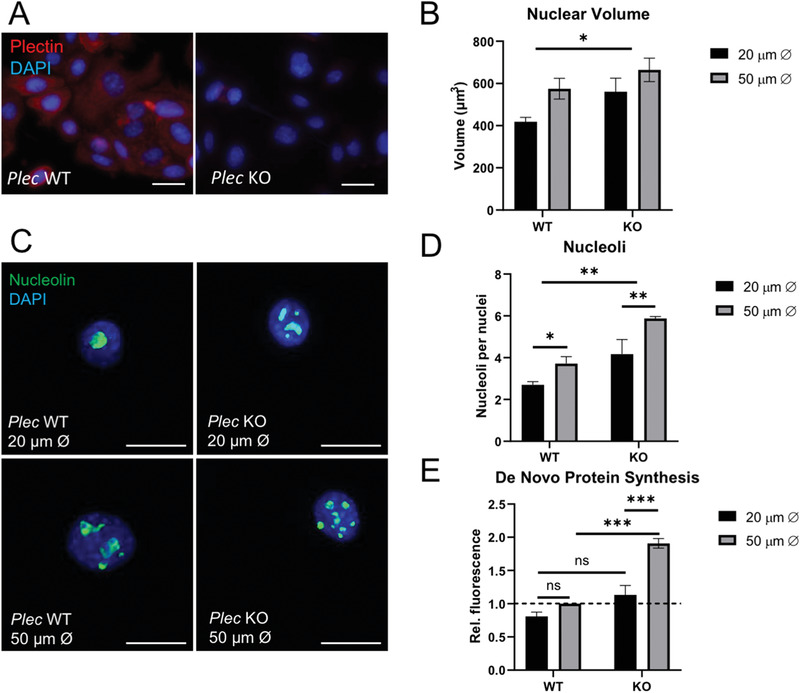
Plectin deficiency promotes increased nuclear volume, nucleoli number, and protein synthesis: A) Representative confocal immunofluorescence images of *Plec* WT and *Plec* KO mouse keratinocytes, stained for nucleus (DAPI, blue) and plectin (red). Scale bars = 20 µm. B) Quantification of nuclear volume of keratinocytes grown on micropatterned surfaces for 24 h. Data represent mean ± SEM, *N* = 4 experiments. n.s: non‐significant. ^*^ *p*‐value < 0.05 (2‐Way ANOVA comparing WT versus KO). C) Representative confocal immunofluorescence images of *Plec* WT and *Plec* KO mouse keratinocytes grown on micropatterned surfaces for 24 h, stained for nucleolin (green) and DAPI (blue). Scale bars = 10 µm. D) Quantification of nucleoli and number and E) de novo protein synthesis levels. Data represent mean ± SEM, *N* = 3 experiments. n.s: non‐significant. ^*^ *p*‐value < 0.05, ^**^ *p*‐value < 0.01, and ^***^ *p*‐value < 0.001 (2‐Way ANOVA, Tukey multiple comparisons test).

To test whether increased nuclear volume reciprocally regulates nucleolar structure, we next took advantage of a mouse keratinocyte cell line lacking the gene *Plec* (KO), which encodes all isoforms of the large cytolinker plectin. Our previous studies have shown that plectin regulates nuclear size,^[^ ^18^ ^]^ and *Plec* KO cells displayed enlarged nuclei compared to wild type (WT) cells (**Figure** **5**A,B). Here, increased nuclear volume in *Plec* KO cells corresponded with an increase in the number of nucleoli and elevated protein synthesis (Figure 5C–E). These results provide further support for a model in which nuclear volume directly regulates nucleolar structure and function.

### 2.5 Chromatin Mobility Modulates Nucleolar Structure and Ribogenesis

**Figure 6:**
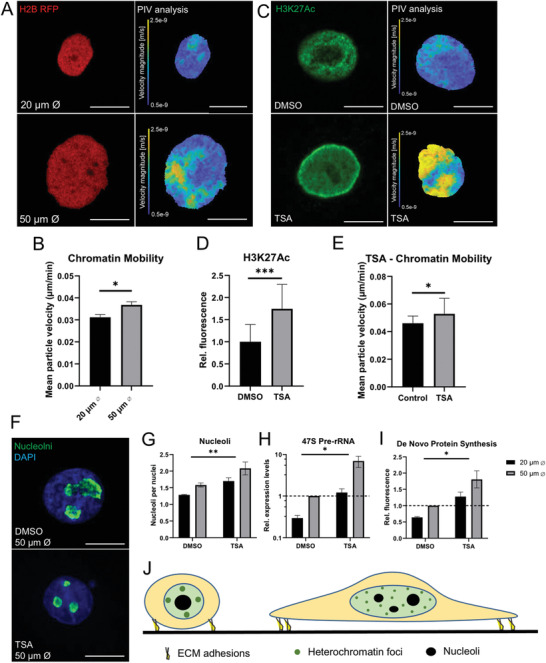
Chromatin mobility modulates nucleolar structure and function: A) Representative live confocal images of HaCaT keratinocytes stably expressing a histone variant H2B‐RFP construct (left—red), grown on micropatterned surfaces for 24 h and particle image velocimetry (PIV) heatmap (right) showing local H2B‐RFP particle velocity over 90 min. Scale bars = 10 µm. B) Quantification of mean HSB particle velocity. Data represent mean ± SEM, *N* = 3 experiments. ^*^ *p*‐value < 0.05 (two‐tail *t*‐test). C) Representative confocal immunofluorescence images of HaCaT keratinocytes treated with DMSO (0.1%) or TSA (200 nm) stained for H3K27Ac (left) and heatmap of PIV analysis of H2B‐RFP particle velocity (right). D) Quantification of normalized integrated intensity of H3K27Ac and E) mean H2B particle velocity. Data represent mean ± SEM, *N* = 3 experiments. ^*^ *p*‐value < 0.05 and ^***^ *p*‐value < 0.001 (two‐tail *t*‐test). F) Represent confocal immunofluorescence images of primary HKs grown on micropatterned surfaces for 24 h stained for nucleus (DAPI, blue) and nucleolin (green). Scale bars = 10 µm. G) Quantification of nucleoli number, H) expression of 47S pre‐ribosomal RNA, and I) de novo protein synthesis levels. Data represent mean ± SEM, *N* = 3 experiments. ^*^ *p*‐value < 0.05 and ^**^ *p*‐value < 0.01 (2‐Way ANOVA, Tukey multiple comparisons test). J) Schematic representation of effects of limited adhesion on nuclear size, heterochromatin condensation, and nucleolar remodeling.

Recent studies on phase separation processes within the nucleus propose that condensation of heterochromatin mechanically excludes liquid‐like droplets and promotes growth and fusion of droplets within softer regions of less dense chromatin.^[^ ^15^ ^]^ This model is consistent with our own observations of condensation of heterochromatin foci and nucleolar remodeling on small micropatterns and potentially explains how reduced nuclear volume alters the chromatin density and drives nucleoli fusion. To test this hypothesis, we first employed a HaCaT keratinocyte line stably expressing an RFP‐tagged histone H2B and analyzed chromatin mobility through particle image velocimetry (PIV) of time‐lapse confocal images (**Figure** **6**A). We observed that the chromatin of keratinocytes cultured on small islands had lower mobility compared to larger islands, indicative of denser and more rigid chromatin (Figure 6B).

We then used the histone deacetylase inhibitor, trichostatin A (TSA), to promote global chromatin de‐condensation and generate a more fluid chromatin state (Figure 6C–E). Primary HKs seeded on micropatterns and treated with TSA exhibited increased nucleoli number, pre‐ribosomal RNA 47S expression, and protein synthesis levels (Figure 6F–I). These findings demonstrate that nucleoli remodeling depends on the global state of the surrounding chromatin and support a model in which liquid–liquid phase separation processes mediate the effects of extracellular adhesive cues on nucleolar structure and function.

### 2.6 Nucleolar Remodeling is Regulated by Diverse Adhesive Cues

**Figure 7:**
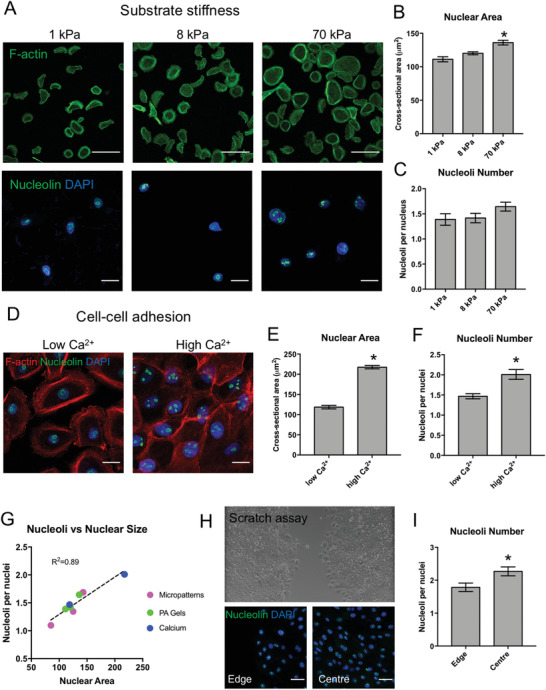
ECM rigidity and cell–cell adhesion regulate nucleolar remodeling: A) Representative fluorescence images of F‐actin (upper panel) and nucleolin and DAPI (lower panel) in HKs grown on PA gels with elastic moduli of 1, 8, and 70 kPa. Scale bars = 100 µm (upper) and 20 µm (lower). B) Quantification of average nuclear cross‐sectional area and C) number of nucleoli per cell. Data represent mean ± SEM, *N* = 3 experiments. ^*^ *p*‐value < 0.05 (ANOVA, Tukey multiple comparisons test). D) Representative fluorescence images of F‐actin, nucleolin, and DAPI in confluent HKs with low (0.06 mm) and high (1.8 mm) Ca^2+^. Scale bar 20 = µm. E) Quantification of average nuclear cross‐sectional area and F) number of nucleoli per cell. Data represent mean ± SEM, *N* = 3 experiments. ^*^ *p*‐value < 0.05 (two tail *t*‐test). G) Correlation and *R* ^2^ (Pearson's coefficient) for nuclear area and nucleoli number per cell combined from HKs cultured on micropatterns, PA gels, or with low or high calcium. Data represent mean of *N* = 3 experiments. H) Representative bright field and fluorescence images of nucleolin in HKs 12 h after scratch wounding. Scale bar = 50 µm (lower panel). I) Quantification of number of nucleoli per cell at the leading edge compared to the center of the monolayer. Data represent mean ± SEM, *N* = 3 experiments. ^*^ *p*‐value < 0.05 (two tail *t*‐test).

Finally, to investigate the wider implications of extracellular adhesive cues on nucleolar remodeling, we examined the influences of ECM rigidity and cell–cell adhesions. When HKs were cultured on collagen coated polyacrylamide (PA) gels with elastic moduli of 1, 8, or 70 kPa,^[^ ^22^ ^]^ a similar relationship between cell spreading, nuclear cross‐sectional area, and nucleoli number could be observed as on the micropatterned substrates (**Figure** **7**A–C). However, the relative effects of ECM rigidity were smaller, and the differences in nucleoli number were not statistically significant. By contrast, confluent HKs cultured in high calcium conditions, which promotes the assembly of cell–cell adhesions, displayed a significant increase in both nuclear area and number of nucleoli compared to cells in low calcium conditions (Figure 7D–F). By combining the analysis of nucleolar remodeling from the micropatterns, PA gels, and different calcium concentrations, a strong correlation between nuclear area and nucleoli number could be observed (Figure 7G), reinforcing the concept that nucleoli number scales with nuclear size.

Given the striking effects of calcium concentration on nucleoli number, we further examined the potential role of cell–cell adhesions within the context of wound healing and re‐epithelialization. In vitro scratch wounds were made on monolayers of HKs, and the number of nucleoli was quantified in cells both at the free leading edge of the wound or multiple cell lengths away from the scratch (Figure 7H). A significant reduction in the average number of nucleoli could be observed at the edge of the scratch compared to confluent cells in the center of the monolayer (Figure 7I). These results further support the role of cell–cell adhesions in regulating nuclear remodeling, whereby the formation of cell–cell adhesions leads to increased nuclear size and a greater number of nucleoli. In addition, these findings provide initial insights into the physiologic significance of nucleolar mechano‐sensing and suggest a potential role in cell migration and wound repair.

## 3 Discussion

The nucleus is a well‐established mechanosensory unit, which converts biophysical stimuli into cell and tissue responses by modulating nuclear architecture and gene expression programs.^[^ ^23^ ^]^ Here, we describe the unique functions of the nucleolus as a mechano‐sensitive organelle, which controls major transcriptional and metabolic processes. In response to altered ECM adhesion, the nucleoli of keratinocytes undergo dramatic remodeling, which scales directly with nuclear volume and is mediated by mechanical linkage to the actin cytoskeleton as well as the physical properties of the surrounding chromatin (Figure 6J). Nucleolar structure also corresponds with downstream changes in the expression of ribosomal genes, rRNA transcription, and protein synthesis, and it is sensitive to a range of different adhesive cues. Thus, the nucleolus is capable of transducing biomechanical cues into key cellular responses.

Recent experimental and computational studies propose a model of nucleoli formation through liquid–liquid phase separation processes in which nucleolar proteins with intrinsically disordered regions self‐associate above a critical concentration.^[^ ^14^, ^24^ ^]^ This process is thermodynamically driven and depends on the properties of both the liquid‐like droplets and surrounding medium.^[^ ^15^ ^]^ Our analysis of chromatin mobility and effects of TSA treatment are consistent with these predictions and suggest that adhesion‐induced changes in chromatin mechanics contributes to the phase separation behavior and fusion of nucleoli. As recent reports have also shown that phase separation processes underlie sub‐nucleolar organization,^[^ ^25^ ^]^ an interesting consideration for future studies will also be how adhesion‐induced changes in nuclear volume influence sub‐compartments of the nucleolus. Likewise, detailed analysis of the interactions between nucleoli and NORs could provide new insights into the reciprocal effects of nucleoli remodeling on genome organization and gene regulation. In our studies, heterochromatin foci number also appeared to be intrinsically linked to nuclear size. Although it has been proposed that heterochromatin foci have phase separation properties, as modulated by HP1‐*α*,^[^ ^26^ ^]^ this view has yet to be proven conclusively,^[^ ^27^ ^]^ and micropatterned substrates may be a useful tool for broader investigation into additional phase separation processes within the cell.

As keratinocytes terminally differentiate in vivo, they detach from the ECM of the basement membrane and undergo well defined changes in nuclear architecture as they move up through the suprabasal layers of the epidermis.^[^ ^28^ ^]^ In parallel with this differentiation process, there is a reduction in nuclear volume and the number of nucleoli in the upper layers of the epidermis.^[^ ^28^ ^]^ These observations are consistent with our in vitro studies and suggest that nuclear volume and nucleoli organization are intrinsically linked in vivo and may have important physiologic roles in the regulation of ribogenesis and protein synthesis. Likewise, our findings from in vitro scratch assays suggest a potential involvement of nucelolar remodeling in wound healing. Given the complexity of the in vivo environment however, further investigation is required to determine the precise roles of different biochemical and mechanical signals. The roles of specific adhesion receptors, such as the *β*1 and *β*4 integrins found in keratinocyte focal adhesions and hemidesmosomes, respectively,^[^ ^29^ ^]^ and cytoskeletal linkers, such talin, vinculin, and paxillin will be of interest in future studies. Along these same lines, biomechanical regulation of nucleolar structure and function may have significant implications in the pathogenesis of genetic skin diseases, such as epidermolysis bullosa. Defects in normal keratinocyte biomechanics or force transmission to the nucleus^[^ ^18^, ^22^, ^30^ ^]^ could potentially impact on nucleoli‐dependent transcriptional and metabolic processes. Thus, nucleolar mechano‐sensing may play an important role in both normal tissue homeostasis and disease states, and further investigation into these mechanisms could lead to the discovery of new therapeutic targets and treatment strategies for such conditions.

## 4 Experimental Section

### Micropatterned Substrate Fabrication

All chemicals were from Sigma Aldrich unless otherwise noted. Master molds for soft lithography were fabricated using photolithography. A silicon wafer (Pi‐Kem) was cleaned using isopropanol then acetone and blown dry with nitrogen gas. The silicon wafer was then coated with SU‐8 2010 (MicroChem) using a spin coater (10 s at 4000 rpm). SU‐8 2010 was then soft‐baked by heating at 100 °C for 5 min before being cooled down at room temperature for 2–3 min. A custom photomask (Micro Litho) containing micropatterned islands was cleaned using nitrogen gas and placed in contact with the SU‐8 coated wafer. The wafer was then exposed to 365 nm UV light (UV‐KUB 6, 100% power for 10 s) before being baked at 100 °C for 5 min. Patterns were developed by submerging the wafer in PGMEA for 2 to 4 min, before being washed with isopropanol and dried with nitrogen gas. Correct pattern dimensions were verified using a profilometer (Profilm3D). Finally, a polydimethylsiloxane (PDMS) stamp was generated by casting 10:1 PDMS:curing agent (Sylgard 184, Farnell) over the micropatterned wafer and incubating overnight at 70 °C before being cut out of the master mold. Each stamp was used for up to 2 months before being discarded.

Micropatterns were generated through micro‐contact printing and surface‐initiated atom transfer radical polymerization, generation poly‐oligo(ethylene glycol methacrylate) polymer brushes. Coverslips (24 mm × 60 mm, 0.17 mm thickness) were initially cleaned using plasma oxidation (Henniker plasma HPT‐200, 100% power, 10 min) then coated with 5 nm of chromium and 15 nm of gold using a thermoevaporator (Moorfields). POEMGA brush polymerization solution was prepared by dissolving 320 mg 2,2′‐bypiridine, 18 mg CuBr2, and 12 mL oligo(ethylene glycol) methyl ether methacrylate (mean molecular weight 300) in 24 mL water and 6 mL ethanol. The solution was degassed using gentle N2 flow under agitation for 30 min. Then, 82 mg of CuCl were added and left to degas for additional 15 min. PDMS stamps were coated with the *ω*‐mercaptoundecylbromoisobutyrate initiator then placed in conformal contact with the gold covered coverslips for 15 s. Stamped coverslips were reacted with the polymerization solution for 15 min. Micropatterned coverslips were removed and washed first with water followed by ethanol and stored for a maximum of 1 month.

Micropatterned substrates were cut using a diamond tipped pen, placed in either 24‐well (≈1 cm^2^) or 6‐well (≈4 cm^2^) before being sterilized using 70% EtOH solution. Micropatterns were coated with 30 µg mL^−1^ sterile solution of Type I Collagen (Corning) in phosphate buffered saline (PBS) overnight at 4 °C. Substrates were washed by dilution thrice using sterile 1 mm HCl solution and twice with PBS before being incubated in FAD medium for a minimum of 30 min at 37 °C before seeding cells.

### Polyacrylamide Gel Synthesis

PA gels were prepared based on previously published protocols.^[^ ^22^ ^]^ Briefly, 13 mm diameter round glass coverslips were first cleaned by three 15‐min sonication cycles in ethanol and left to dry overnight. A silane coating was then applied by immersing the coverslips in a 5% v/v 3‐aminopropyltrimethylsiloxane in water solution for 30 min under agitation. After 6 washes with water, coverslips were incubated with a 0.5% v/v glutaraldehyde solution in PBS for 15 min. Coverslips were washed three times with deionized water and left to dry over‐night. In order to prepare PA gels with varied rigidities, different stock solutions were prepared with a ratio variation between PA and bis‐acrylamide.^[^ ^22^ ^]^ These stock solutions were de‐gassed for 5 min at room temperature and further stored at 4 °C until use. To create a thin layer of PA on the coverslip surface, 1 mL of each stock solution was mixed with 4 µL of TEMED and 6 µL of 10% v/v ammonium persulfate in water. Drops of 10 µL of the final mixture were placed on a Sigmacote‐coated microscope slide and then covered by the silane‐coated coverslips. Gels were allowed to cross‐link for 5 min at room temperature. After this period, each slide was immersed in a 50 mm HEPES solution in water. Once hydrated, the gels were gently peeled off the microscope slide and stored at 4 °C in 50 mm HEPES solution until use.

To promote cell attachment PA gels were functionalized with type I collagen. PA gels were first activated by incubation with 0.2 mg mL^−1^ of the cross‐linker Sulfo‐SANPAH (BioVision) and exposure to 365 nm UV light for 5 min. Gels were washed twice with deionized water and the reaction was repeated a second time. Gels were then washed twice with PBS, incubated with 50 µg mL^−1^ of collagen for 3 h at room temperature, and washed twice more with PBS. Prior to cell seeding, gels were sterilized by exposure to 254 nm UV light for 3 min in a UVP 95‐0339‐02 cross‐linking drawer (John Morris Group) followed by two sterile PBS washes, a brief rinse with 70% v/v ethanol and two final washes with PBS. PA substrates were incubated with cell culture media for 30 min immediately before cell seeding.

### Cell Culture

All reagents were from ThermoFisher unless otherwise noted. The J2 clone of Swiss 3T3 cells were cultured in High glucose DMEM with L‐glutamine (Gibco) and supplemented with 1% Pen/Strep (Gibco) and 10% FBS (Biosera). Cells were passaged bi‐weekly, at confluence, and replated at a 1:10 dilution. Cells were maintained until the 25th passage before being discarded. All cell lines were maintained in 5% CO_2_ at 37 °C.

Primary HKs were maintained on a feeder layer of inactivated J2 cells as previously described.^[^ ^31^ ^]^ Confluent J2 3T3s were treated with 0.4 mg mL^−1^ Mitomycin C for 3 h and replated at 3E6 per T75 flask the day before primary keratinocyte seeding. Primary keratinocytes were seeded onto the J2 feeder layer at a density of 5E5 keratinocytes per T75 and co‐cultured in FAD medium, composed of 3 parts DMEM (Gibco) and 1 part F12 (Gibco) supplemented with 1% Pen/Strep (Gibco), 10% FBS, 0.18 mm adenine, 1 nm cholera toxin, 10 ng mL^−1^ epidermal growth factor (Peprotech), 0.5 µg mL^−1^ hydrocortisone, and 5 µg mL^−1^ insulin. When passaging co‐cultures, medium was aspirated off and cells were washed thrice with pre‐warmed Versene solution (Gibco). Each wash was accompanied with tapping, to detach Mitomycin C‐treated J2s. Remaining HKs were then trypsinized using pre‐warmed 0.10% trypsin in 0.48 mm EDTA solution (Gibco) for 5 min. Primary keratinocytes used were from existing stocks of cells extracted from neonatal foreskin samples. All samples were obtained following routine circumcision procedures at the Royal London Hospital, under the ethical reference number LREC 08/H0704/65. Cells were used from passage 2 to passage 8 before being discarded.

HaCaT keratinocytes were cultured in high glucose DMEM with L‐glutamine (Gibco) and supplemented with 1% Pen/Strep (Gibco) and 10% FBS (Biosera). They were passaged bi‐weekly and split in a 1:10 dilution. Stably transfected HaCaTs expressing H2B‐RFP were selected using Neomycin (500 µg mL^−1^) and sorted by flow cytometry to select the higher‐expressing population.

*Plec* KO and WT mouse keratinocytes were provided by Prof. Gerhard Wiche. Cell cultures were established from Plec^−/−^/p53^−/−^ and Plec^+/+^/p53^−/−^ mice, as described elsewhere.^[^ ^32^ ^]^ They were cultured in EpiLife medium (Gibco) supplemented with 1% Pen/Strep (Gibco) and KSFM supplements (5 mg cL^−1^ Bovine Pituitary Extract, 0.5 µg cL^−1^ EGF, Human Recombinant). Cell culture flasks were coated with type I collage before seeding cells. Cells were passaged once weekly used from passage 12 to passage 20 before being discarded.

After passaging and resuspension, keratinocytes were seeded onto micropatterned substrates at defined densities of 2E5 cells mL^−1^ for 20 µm islands and 4E4 cells mL^−1^ for 50 µm islands in their required media. Cells were left to attach for 1–2 h before excess cells were washed off three times with fresh media and the substrates were transferred into new wells containing fresh media. For PA gel studies, HKs were seeded onto the gels at density 2E4 cells per gel and cultured for 24 h. For calcium studies, HKs were seeded onto 13 mm collagen‐coated glass coverslips at a density of 5E4 cells per coverslip and allowed adhere overnight in low calcium chloride (0.06 mm) Epilife medium (ThermoFisher). Cells were switched to Epilife supplemented with low or high (1.8 mm) calcium chloride and cultured for an additional 24 h. For the scratch assays, HKs were seeded onto collagen‐coated glass bottom dishes (Corning) at a density of 2E4 cells cm^−2^ and cultured overnight in Epilife with high calcium. Scratch wounds were created with a 10 µL pipet tip and cells were fixed for analysis 12 h after wounding.

### Immunofluorescence Staining

Cells were rinsed with PBS cells and fixed using 4% paraformaldehyde in PBS for 10 min before being washed twice in PBS. Cells were permeabilized using 0.2% Triton X100 in PBS for 5 min. For nucleolin staining, 0.1% Triton X100 for 15 min was used instead. Cells were then blocked using 10% FBS and 0.25% fish skin gelatin in PBS for 1 h at room temperature. Primary antibodies in blocking solution were then applied and left for incubation overnight at 4 °C. Samples were washed in PBS before applying secondary antibodies and DAPI (Biotium) for 1 h at room temperature. Finally, samples were washed in PBS followed by H2O before being mounted onto microscope slides using 5 µL of Mowiol. Antibodies used are available in Table S2, Supporting Information.

### Fluorescence Imaging and Processing

Epifluorescence images were acquired using a Leica DM4000 Microscope and 40× objective. Confocal images were acquired using a Zeiss 880 Laser Scanning Confocal Microscope. Confocal images were acquired using AiryScanFast2D configuration, Plan‐Apochromat 63×/1.4 Oil DIC M27 objective, 2.3× digital zoom. Images were 1024 × 1024 pixels, 17.4522 pixels per micron. The middle section of nuclei was determined using Z‐stack range indicator. Z‐stack slices were spaced by 0.5 µm.

Primary analysis of images was performed using ImageJ software (v.1.51w, National Institutes of Health). Images had their background removed first (rolling ball radius 50 pixels), before delimiting regions of interest with magic wand tool and measuring intensity. DAPI levels were used to normalize all intensity measurements, before being normalized to control condition (50 µm islands). Morphological analysis was performed by first generating a binary image. If analyzing Z‐stacks, the middle section was used to calculate the threshold. Then holes in the binary image were filled and the ask area was then measured. For nuclear volumes the 3D geometrical measure plugin was used.

Spatial distribution of the nuclear lamina and histone modifications was analyzed using CellProfiler software (v.2.2.0, www.cellprofiler.org, BROAD Institute). DAPI images were used to generate a mask and radial intensity distribution within the masked region of interest was measured by dividing the nuclear area in 20 bins of equal radial distance and calculating the mean intensity for each bin.

Heterochromatin foci analysis was performed using the InCell Developer Toolbox (v.1.9.2 build 2415, GE Healthcare). Nuclei masks were obtained by performing intensity segmentation on DAPI images and subsequently passed through a sieve to discriminate objects based on size (>3000 pixels and <250 000 pixels). Finally, the mask was dilated (kernel size 3) and eroded (kernel size 11) to remove part of the nuclear periphery. Foci were determined by performing object segmentation on H3K9me3 images with a small kernel size of 7 and sensitivity of 40. A sieve was used to discriminate foci (>10 pixels and <1000 pixels), and objects that did not share more than 95% boundary with mask obtained from DAPI staining were removed.

### Nucleoli Live Imaging

HaCaT cells were transfected with 3 µg of GFP‐Nucleolin plasmid (Addgene, plasmid #28176) using JetPRIME transfection reagent (Polyplus) 24 h before seeding cells on micropatterned islands. Micropatterned islands were attached onto a bottomless round confocal dish (VWR) using silicone adhesive sealant (Momentive). 45 min after seeding excess cells were washed off with fresh medium and attached cells were imaged with a Zeiss 880 Laser Scanning Confocal Microscope with incubator chamber attached to maintain 5% CO_2_ and 37 °C. Five cells per island size were imaged per experiment. Z‐stacks were taken every 15 min for a total of 3 h (12 stills per cell).

### RNA Sequencing

Primary HKs were seeded onto micropatterned substrates as previously described. After 4 or 24 h samples were washed with PBS before being lysed with RLT Plus Lysis Buffer (Qiagen). RNA extraction was performed using RNeasy Plus Micro Kit (Qiagen) and RNA eluted in RNAse‐free water. Paired‐end RNA sequencing was performed using the NextSeq 500 with 40 million reads per sample and 75 bp read length (QMUL Genome Centre). FASTQ files were uploaded to Galaxy (https://usegalaxy.org/), aligned to the human genome (reference hg19) using HISAT2, and exported to BAM files. BAM files were then analyzed in SeqMonk (Babraham Bioinformatics, version 1.45.0). RNA‐Seq QC Plot was used to verify correct alignment. Differential expression of genes was performed by DeSeq2 with raw counts between conditions (alpha set at 0.05 level). Lists of differentially expressed genes were then uploaded to the Gene Ontology Resource (http://geneontology.org/) to further analyze affected pathways.

### RT‐qPCR

Cells were washed once with PBS and subsequently lysed with RLT Plus lysis buffer (Qiagen) before RNA extraction. RNA was extracted from samples using RNeasy Plus Micro kit (Qiagen) as per manufacturer's instructions. RNA was then reverse transcribed using LunaScript RT SuperMix kit (NEB). RT‐qPCR was performed using qPCRBIO SyGreen Mix (PCR Biosystems). All probes were done in duplicates, and no‐template controls were introduced for all probes. RT‐qPCR was performed in a StepOnePlus RT‐qPCR System, and data were analyzed using StepOne Software v2.3 (Applied Biosystems). ∆*Ct* was calculated from averaged *Ct* of duplicates (*Ct*_target_) and compared to reference housekeeping gene (*Ct*_ref_). qPCR probe list is available as Table S1, Supporting Information.

### De Novo Protein Synthesis Assay

Nascent protein levels were determined using Click‐iT HPG Alexa Fluor 488 Protein Synthesis Assay Kit (ThermoFisher). Cell media was switched with methionine‐free media supplemented with L‐homopropargylglycine. After 30 min cells were washed once with PBS, fixed and permeabilized as per normal IF protocol. Nascent protein was then labeled using 1X Click‐iT HPG reaction cocktail (ThermoFisher) for 30 min at room temperature, before being aspirated off and cells were washed with Click‐iT reaction rinse buffer (ThermoFisher). Cells were subsequently labeled for their nucleus using HCS NuclearMask Blue Stain and mounted onto coverslips using Mowiol. Samples were then imaged by epifluorescence microscopy. Nascent levels of protein were assessed by measuring Alexa‐488 nm fluorescence levels.

### Retinoic Acid Reporter Assay

Primary HKs were transfected with 0.5 µg Thymidine Kinase—Renilla Luciferase control (Addgene plasmid #16539) and 0.5 µg RA Response Element—Firefly Luciferase (Addgene plasmid #13458) per 1E5 cells using JetPRIME transfection reagent (Polyplus, 114‐07) according to the manufacturer's instructions. Transfected cells were seeded onto micropatterned islands. Positive controls were treated with 1 µm all‐trans‐RA (ATRA) and negative controls were not transfected. Cells were lysed after 24 h on micropatterns, and luciferase activity was measured using the Dual Luciferase Assay System (Promega). Each sample was analyzed in triplicate with a CLARIOstar plate reader (BMG Labtech), and the RARE‐Luciferase signal was normalized the internal Renilla control signal.

### siRNA Treatment

Primary HK (1E6 cells) were seeded into a type I collagen coated T25 culture flask and cultured in KSFM for 24 h. Cells were transfected with 20 nm siRNA and 8 µL JetPRIME transfection reagent (Polyplus, 114‐07). siRNAs were: Silencer Select *LMNA* (s8221), *SYNE2* (s23328), and negative control (4390843). Transfection was repeated after 48 h, and 96 h after initial seeding cells were detached from cell culture flask and seeded onto micropatterned islands as previously described.

### H2B‐RFP Live Imaging and PIV Analysis

HaCaT cells stably expressing H2B‐RFP construct were seeded onto either unmodified round confocal dish (VWR) or modified with micropatterned substrates as described above. After 1 h, cells were treated with DMSO (0.1%) or TSA (200 nm) and cultured for 24 h. Time‐lapse imaging was performed using a Zeiss LSM 880, and Z‐stack images of cells were acquired every 3 min for 90 min. Drift correction was performed with ImageJ, and central frames were analyzed by PIV using PIVLab (Matlab). Interrogation passes were as follows: 1st pass 32 pixels, 2nd pass 16 pixels, 3rd pass 8 pixels, and 4th pass 4 pixels. Mean velocity magnitude was calculated across all frames for each nucleus.

### Western Blot Analysis

Cells were washed using cold PBS (Gibco) then adherent cells scraped off and lysed using RIPA buffer (ThermoFisher) supplemented with phosphatase inhibitor (phosphoSTOP, Roche) and Protease Inhibitor Cocktail (Sigma). Lysates were then sonicated (30 s intervals for 5 min on ice) with a Diagenode Bioruptor, centrifuged (2500 RPM, 30 min at 4 °C), and supernatant collected. Total protein concentration was quantified using the Pierce BCA assay (ThermoFisher). Samples were mixed with 4× sample buffer (ThermoFisher) and *β*‐mercaptoethanol and heated at 95 °C for 5 min to denature. Protein was separated on 4–15% gradient Mini‐Protean gels (Bio‐Rad) for 90 min at 100 V and then then transferred onto a nitrocellulose membrane (GE Healthcare) at 300 mA for 1 h on ice. Protein loading and transfer was assessed by Ponceau staining before rinsing with Tris‐buffered saline with 0.5% Tween‐20 (TBS‐T) blocking in 5% non‐fat dry milk (Marvel) in TBS‐T for 1 h. Membranes were then incubated with primary antibody in 5% milk/TBS‐T overnight at 4 °C. Membranes were washed three times with TBS‐T for 5 min per wash, before secondary antibody incubation in milk/TBS‐T for 1 h at room temperature. Membranes washed three times with TBS‐T and incubated with HRP substrate peroxide/HRP substrate luminol reagent (50:50, Millipore) for 30 s. Excess reagent was removed, and membranes were developed using ChemiDoc Gel Imaging System (Bio‐Rad).

### Statistical Analysis

Statistical analysis of data was performed using Prism (version 8.4.3, GraphPad). All data represent the mean of at least three independent experiments. For quantification of nucleoli numbers and protein synthesis, at least 30 cells were analyzed per experiment, and for 3D analysis of nuclear volume and chromatin remodeling, at least 10 cells were analyzed per experiment. When comparing two conditions for normal data, significance was investigated through unpaired *t*‐tests. When comparing three conditions or more for normal data, significance was investigated using 1‐ or 2‐way ANOVAs. Multiple comparisons were investigated through Tukey's multiple comparisons test when comparing four conditions. Radial intensity profiles were analyzed using the Kolmogorov–Smirnov test. Statistical significance was determined by *p* < 0.05 for all tests.

## Conflict of Interest

The authors declare no conflict of interest.

## Supporting information

Supporting Information

## Data Availability

The data that support the findings of this study are available from the corresponding author upon reasonable request.
