# Supplementary material for: Extracellular Adhesive Cues Physically Define Nucleolar Structure and Function
Source: Adv Sci (Weinh). 2022 Feb 5;9(10):2105545. doi: 10.1002/advs.202105545 (PMC8981897; doi:10.1002/advs.202105545)
Supplement: Supplementary file 1 — Supporting Information [file ADVS-9-2105545-s001.pdf]

ADVANCED SCIENCE
Open Access

Supporting Information

for *Adv. Sci.*, DOI 10.1002/advs.202105545

Extracellular Adhesive Cues Physically Define Nucleolar Structure and Function

*Oscar J. Pundel, Liisa M. Blowes and John T. Connelly**

ADVANCED SCIENCE
Open Access

Supporting Information

for *Adv. Sci.*, DOI: 10.1002/advs.202105545

Extracellular adhesive cues physically define nucleolar structure and function

*Oscar J. Pundel, Liisa M. Blowes, and John T. Connelly**

Supporting Information

Extracellular adhesive cues physically define nucleolar structure and function

*Oscar J. Pundel, Liisa M. Blowes, and John T. Connelly**

Table S1. qPCR primer list:

Gene	Forward (5' to 3')	Reverse (5' to 3')
GAPDH	ACCCACTCCTCCACCTTTG	CTCTTGTGCTCTTGCTGGG
B2M	ATGGAGGTTTGAAGATGCC	CTAAGTTGCCAGCCCTCCT
7SK	GAGGGCGATCTGGCTGCGA CAT	ACATGGAGCGGTGAGGGAG GAA
H3.1	TCCGCCGTTATCAGAAGTCC	GTGTCCTCAAATAGCCCTAC C
BRCA1	TATCACCACTGAATCTCTAC CG	GACCTCAAACTCTGAGATC CAC
ATR	GAACACCACTGAGAAGCGT G	CCACATGGCTCCACATGCA A
ATM	TGCTGACAATCATCACCAA GTTC	TCTCCCTTCGTGTCCTGGAA
RARG	GCCCTTCTGTACTGTCCATG T	AGAAGCCCAATGGATAGGG TA
CRABPII	TGATGAGGAAGATCGCTGT G	TTCCACTCTCCCATTTCACC
NCL	TGTCAGCCCTGTTCCATGTC	GCTTGCCTCATAGGAGACC C
RPL36	CTGGTGCCAGACGTGTTACT	TGGAAACACGCACTAAGCC A
RRP1B	AAGAACACGCCCCACTTCA A	AGCAGAAATGTCCTCCGCA A
H47S pre-rRNA	GCTGACACGCTGTCCTCTG	TCGGACGCGCGAGAGAAC
45S pre-rRNA (Mouse)	GTTCCCGTGTTTTTCCGCTC	CATCGGAGAGCATCAGCCA T
RPL36 (Mouse)	CATCGGAGAGCATCAGCCA T	ATCATGTCCCGCACGAACTT

Table S2. Antibody list:

**Antibody**	Species	Fixation Method	Dilution (IF/WB)	Manufacturer	Reference
Lamin A/C	Mouse	PFA 4%	1:200/1:1000	Santa Cruz	sc-7292
Lamin B1	Rabbit	PFA 4%	1:500	Abcam	ab16048
pSer22 Lamin A	Rabbit	PFA 4%	1:500	ThermoFisher	PA517113
LAP2α	Rabbit	PFA 4%	1:500	Abcam	ab5162
Nesprin 2	Rabbit	N.A	/1:1000	Abcam	ab103020
H3K27me3	Rabbit	PFA 4%	1:200	Millipore	07-449
H3K27Ac	Rabbit	PFA 4%	1:500	Abcam	ab4729
H3K9me3	Rabbit	PFA 4%	1:400	Abcam	ab8898
H3K4me3	Rabbit	PFA 4%	1:200	Millipore	07-473
Transglutaminase 1 (BC1)	Mouse	PFA 4%	1:1000	CRUK	N.A
GAPDH	Mouse	N.A	/1:20000	Millipore	MAB374
Nucleolin	Rabbit	PFA 4%	1:400	Abcam	ab22758
Collagen I	Mouse	PFA 4%	1:2000	Abcam	ab90395
γH2AX	Mouse	PFA 4%	1:1000	Millipore	5636
53BP1	Rabbit	PFA 4%	1:200	Bethyl	A300-273A
Keratin 14 (LL002)	Rat	PFA 4%	1:500	eBioscience	CBL197
Pan Keratin (H-240)	Rabbit	PFA 4%	1:500	Santa Cruz	sc-15367
Plectin (10F6)	Mouse	MeOH	1:200	Santa Cruz	sc-33649
Alexa 488/555 Anti-Mouse	Donkey	-	1:1000	Molecular Probes	
Alexa 488/568 Anti-Rabbit	Goat	-	1:1000	Molecular Probes	
Anti-mouse HRP	-	-	1:5000	Dako	
Anti-rabbit HRP	-	-	1:5000	Dako	
DAPI	Dye	-	1:1000	Molecular Probes	
Phalloidin 488	Dye	-	1:200	Molecular Probes	

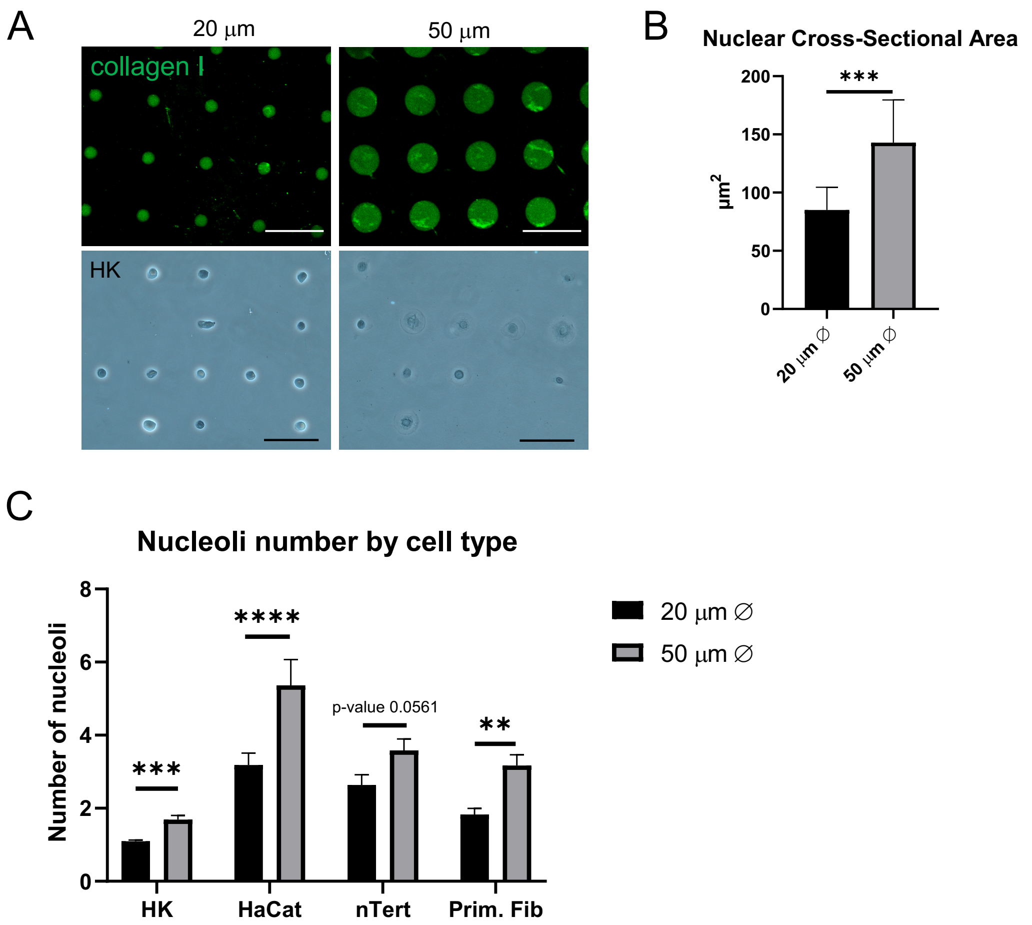

Figure S1. Effects of cell-ECM adhesion nuclear morphology and nucleoli number: (A) Representative images collagen micropatterns and brightfield images of cells on 20 µm and 50 µm islands. Scale bars = 100 µm. (B) Quantification of nuclear cross-sectional area from confocal images of DAPI staining of primary HKs grown on micropatterned surfaces after 24 hours. Data represent mean ± SD, N = 5 experiments.***: p-value < 0.001 (two-tail t-test). (B) Quantification of nucleoli number of primary keratinocytes (HK), immortalized keratinocyte lines (HaCaT and NTERT), and primary dermal fibroblasts. grown on micropatterned surfaces for 24 hours as determined by epifluorescence imaging of nucleolin. Data represent mean ± SD, N = 30 cells. **: p-value < 0.01. ***: p-value < 0.001. ****: p-value < 0.0001 (two-tail t-test).

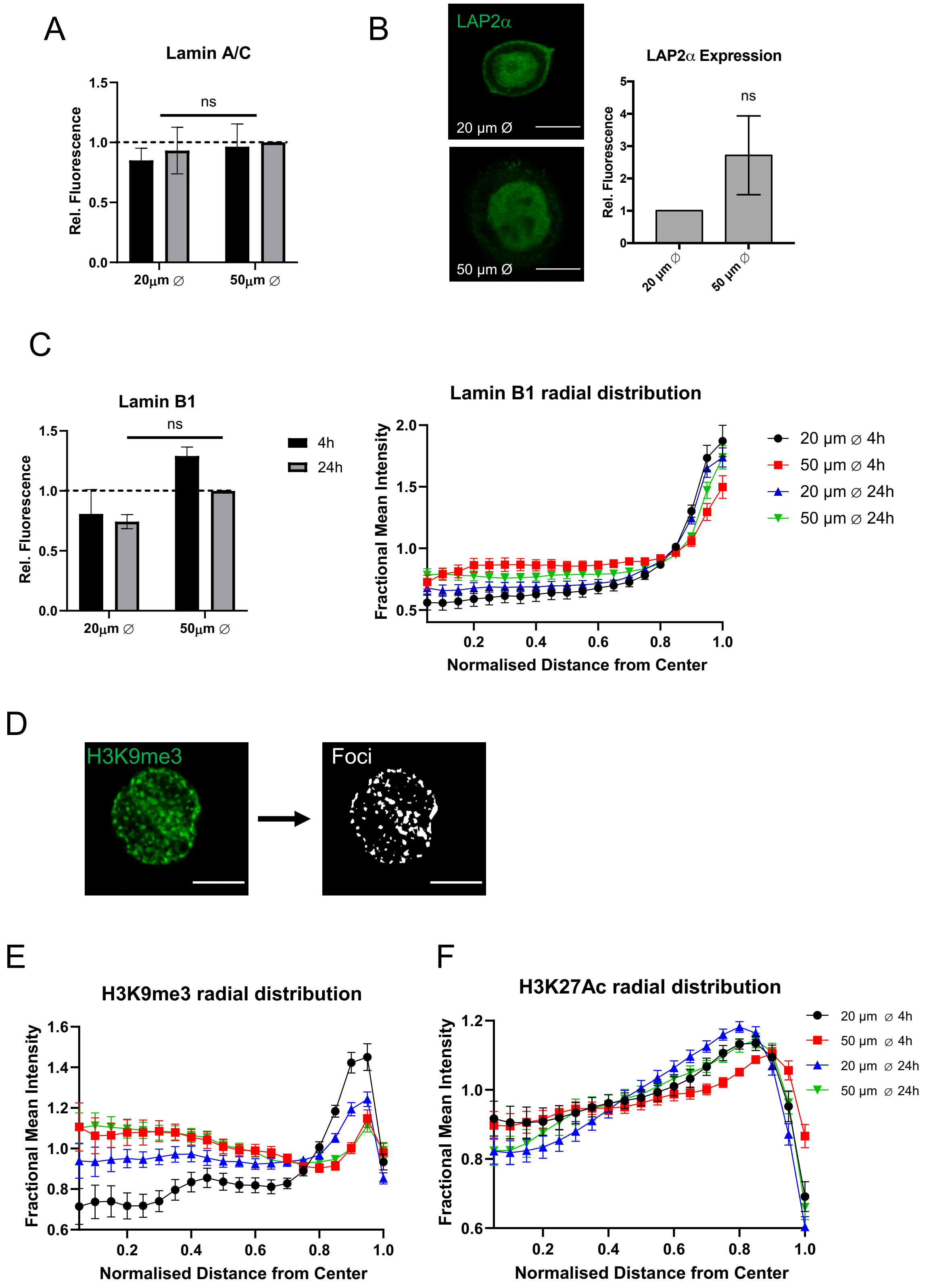
A
Lamin A/C
ns
Rel. Fluorescence
1.5
1.0
0.5
0.0
20µm ∅
50µm ∅
B
LAP2α
20 µm Ø
50 µm Ø
LAP2α Expression
ns
Rel. Fluorescence
20 µm ∅
50 µm ∅
C
Lamin B1
ns
4h
24h
Lamin B1 radial distribution
Fractional Mean Intensity
Normalised Distance from Center
20 µm ∅ 4h
50 µm ∅ 4h
20 µm ∅ 24h
50 µm ∅ 24h
D
H3K9me3
Foci
E
H3K9me3 radial distribution
F
H3K27Ac radial distribution

Figure S2. Effects of cell-ECM adhesion on lamin A/C overall levels, lamin B1 radial distribution, and chromatin radial distribution: (A) Quantification of normalized integrated intensity of lamin A/C. Data represent mean ± SEM, N = 3 experiments. n.s: non-significant. (2-Way ANOVA, Tukey multiple comparisons test). (B) Representative images and quantification of LAP2α expression in HKs cultured on micropatterned surfaces for 24h. Data represent mean ± SEM, N = 3 experiments. n.s: non-significant. (two tailed t-test). (C) Lamin B1 quantification of immunofluorescence images of HKs grown on micropatterned surfaces. Data represent mean ± SEM, N = 3 experiments. n.s: non-significant. (2-Way ANOVA, Tukey multiple comparisons test). Radial distribution of lamin B1 cytoskeletal protein plotted as radial mean fractional intensity (mean intensity = 1). Data represent mean ± SEM of 3 independent experiments. (D) Representative image of H3K9me3 foci analysis showing initial image and detected foci. Scale bar is 10 µm (E) Radial distribution of H3K9me3 and (F) H3K27Ac chromatin markers plotted as radial mean fractional intensity (mean intensity = 1). Data represent mean ± SEM of 3 independent experiments.

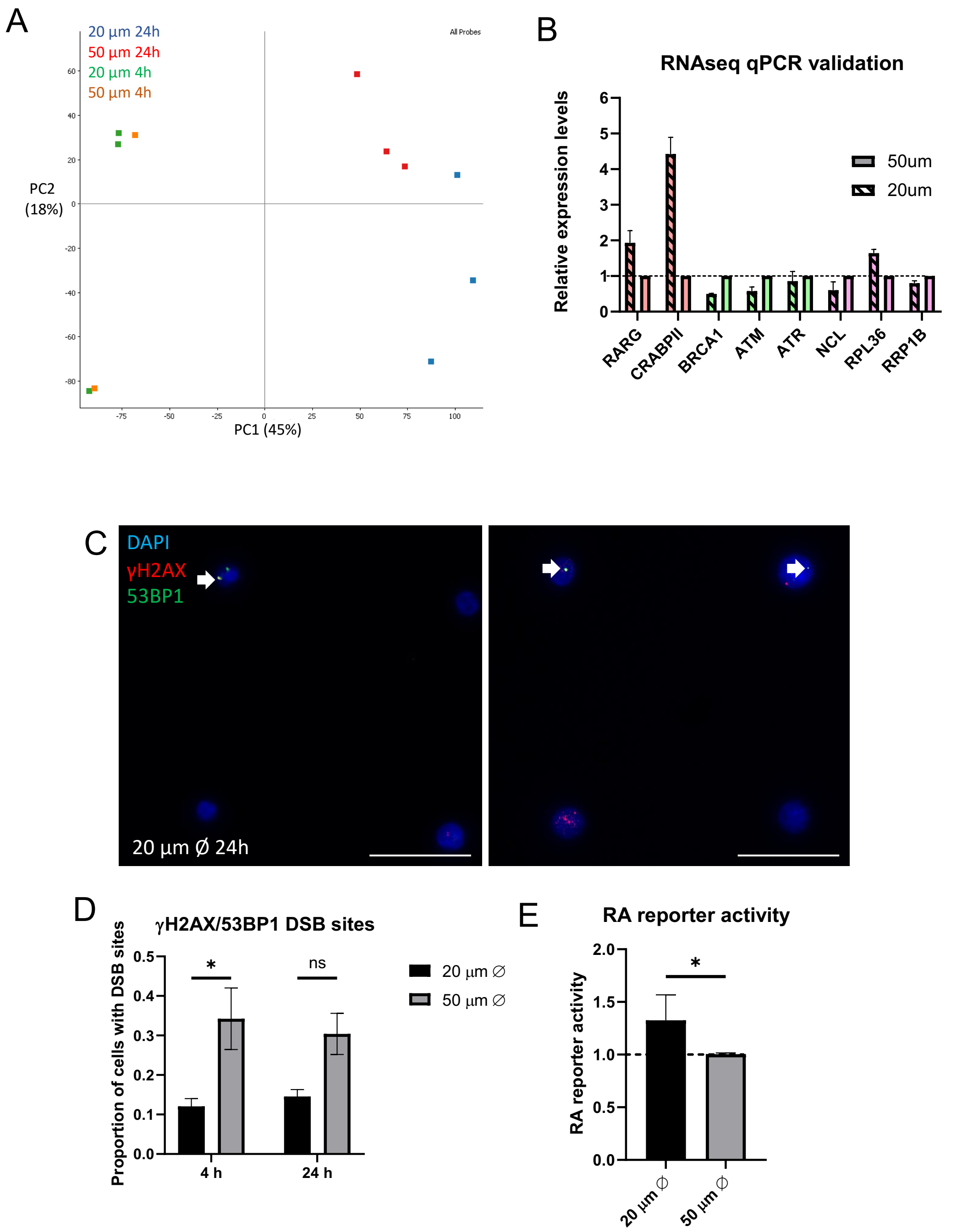

Figure S3. Cell-ECM adhesion regulates specific transcriptional responses with associated phenotypic changes: (A) Principal components plot of RNAseq samples. (B) Validation of RNAseq data by quantification of RNA expression by qPCR of selected genes. Data represent mean ± SEM, N = 3 experiments. (C) Representative epifluorescence images of primary HKs grown on micropatterned surfaces for 24 hours and stained for nucleus (DAPI, blue), phosphorylated histone variant γH2AX (red) and 53BP1 (green). Scale bars = 50 µm. (D) Quantification of HKs undergoing double strand break (DBS) repair shown as proportion of cells with colocalizing foci of γH2AX and 53BP1. Arrows indicate examples of colocalizing foci. Data represent mean ± SEM, N = 3 experiments. n.s: non-significant. *: p-value < 0.05 (2-Way ANOVA, Tukey multiple comparisons test). (E) Quantification of Retinoic Acid Reporter Element activity through luciferase assay. Data represent mean ± SEM, N = 3 experiments. *: p-value < 0.05 (two-tail t-test).
